# ADMA mediates gastric cancer cell migration and invasion via Wnt/β-catenin signaling pathway

**DOI:** 10.1007/s12094-020-02422-7

**Published:** 2020-06-30

**Authors:** Q. Guo, J. Xu, Z. Huang, Q. Yao, F. Chen, H. Liu, Z. Zhang, J. Lin

**Affiliations:** Department of Oncological Surgery, The Second Affiliated Hospital of Fujian Medical University, 34 Zhongshanbei Road, Quanzhou, 362000 Fujian China

**Keywords:** GC, Gastric cancer, ADMA, Asymmetric dimethylarginine, EMT, Epithelial-mesenchymal transition

## Abstract

**Objective:**

To explore the role of ADMA in gastric cancer.

**Methods:**

The specimens of 115 gastric cancer patients were analyzed by ELISA and survival analysis. Functional assays were used to assess the effects of ADMA on gastric cancer cells. Experiments were conducted to detect the signaling pathway induced by ADMA in GC.

**Results:**

Gastric cancer patients with high ADMA levels had poor prognosis and low survival rate. Furthermore, high level of ADMA did not affect the proliferation while promoted the migration and invasion of gastric cancer cell. Moreover, ADMA enhanced the epithelial–mesenchymal transition (EMT). Importantly, ADMA positively regulated β-catenin expression in GC and promoted GC migration and invasion via Wnt/β-catenin pathway.

**Conclusions:**

ADMA regulates gastric cancer cell migration and invasion via Wnt/β-catenin signaling pathway and which may be applied to clinical practice as a diagnostic and prognostic biomarker.

**Electronic supplementary material:**

The online version of this article (10.1007/s12094-020-02422-7) contains supplementary material, which is available to authorized users.

## Introduction

Gastric cancer (GC) is one of the most common malignant tumors of the digestive tract. According to the National Central Cancer Registry of China, there were about 679,000 new cases of gastric cancer in China in 2015, and about 498,000 deaths, with the mortality rate of GC being the second highest among all malignant tumors. The early detection rate of gastric cancer in China is low [1]. Some patients have lymph node metastasis at the first diagnosis, and even distant organ metastasis, which leads to the loss of surgical cure and affects the prognosis and survival rate of patients [2]. Despite the advancement of modern medical technology, the continuous development of endoscopic techniques and the remarkable progress of immunotherapy, the current treatment methods have limited efficacy in the treatment of gastric cancer, with the mortality rate of gastric cancer in China remaining high [3–6]. The incidence of gastric cancer in China accounts for about 42.6% of the world and the death of gastric cancer accounts for about 45.0% [7]. Therefore, identification of novel biomolecules and signaling pathways may provide potential therapeutic strategies for GC.

A healthy adult produces 300 µmol (~ 60 mg) of ADMA per day [8]. ADMA is mainly synthesized by protein arginine methyltransferases (PRMTs). PRMTs use S-adenosylmethionine as a methyl donor to transfer methyl groups to the nitrogen atom of the guanidinium group of arginine, catalyzing the methylation of arginine residues [8–10]. About 80% of ADMA are degraded into citrulline and dimethylamine by DDAH1 and about 50 µmol of ADMA is excreted by kidney as a prototype [11, 12]. It has been reported that ADMA serum levels were high in a variety of patients with tumor, including lung cancer, hematopoietic tumor, breast cancer, gastric cancer, esophageal cancer and colon cancer, but its role in tumor development is still unclear [13, 14]. Some studies in colon cancer indicated that ADMA treatment attenuated cell death in LoVo cells induced by serum starvation (SS) and doxorubicin hydrochloride [15]. Some studies in pheochromocytoma cells have suggested that ADMA can reduce the glutamate-induced cytotoxicity, apoptosis and Caspase-3 activation and reverse the down-regulation of glutamate-induced bcl-2 expression [16]. Also, it has been reported that ADMA levels may be increased in non-oncologic processes such as radial artery spasm, coronary artery disease and so on [17, 18]. Although, ADMA plays a significant role in promoting tumor progression in colon cancer and esophageal cancer, the role of ADMA in gastric cancer has not been well investigated. Additionally, we have found that DDAH1 inhibitor (PD404182) promoted the epithelial–mesenchymal transition(EMT) progression as well as the migration and invasion via Wnt signaling pathway in GC cells [19]. ADMA are mainly degraded by DDAH1,and DDAH1 inhibitor treatment led to accumulation of ADMA in vitro [20]. Therefore, to determine whether the ADMA promote GC migration and invasion via Wnt signaling pathway as well, we performed a series of experiments with different concentrations of ADMA to support our guess.

A slice of evidences stated that the Wnt/β-catenin pathway was one of the key factors inducing metastasis [21, 22]. Wnt proteins constitute a large family of secreted lipid modified glycoproteins. The Wnt family is implicated in a variety of cellular processes, such as proliferation, apoptosis, differentiation, and migration [23]. A great quantity of evidences have suggested that the canonical Wnt/β-catenin pathway plays a critical role in inducing cancer steam cell to undergo EMT. And EMT is a highly conserved and fundamental process that is critical for embryogenesis and some other pathophysiological processes, particularly tumor genesis and progression. Aberrant gastric EMT activation could endow gastric epithelial cells with increased mesenchymal characteristics and less epithelial features, and promote cancer cell stemness, initiation, invasion, metastasis and chemo-resistance with cellular adhesion molecules repressed, which allows tumor cells to disseminate and spread throughout the body. EMT is modulated by diverse micro-environmental, membrane, and intracellular cues, and could be triggered by various overexpressed transcription factors, which are downstream of several vital cross-talking signaling pathways including Wnt/β-catenin [21, 24, 25]. Therefore, our work was aim to elucidate the relationship between ADMA and EMT in GC dissemination. The result may further provide insights into the potential and mechanism of ADMA in promoting GC invasion and metastasis.

## Materials and methods

### Cell lines and clinical samples

Six human GC cell lines (NCI-N87, MKN74, AGS, NUGC3, MGC803, HGC-27) were obtained from the Type Culture Collection of the Chinese Academy of Sciences (Shanghai, China). All cell lines were maintained in RPMI-1640 supplemented with 10% FBS except AGS in DMEM/Ham’s F12 medium and incubated at an atmosphere containing 5%CO2 at 37 °C. Gastric adenocarcinoma patients (*n* = 115) were randomly enrolled in between January 2013 and December 2014 at the Department of General Surgery of the Second Affiliated Hospital of Fujian Medical University. All patients were diagnosed pathologically according to the American Joint Committee on Cancer (AJCC) criteria [26]. No patient had received chemotherapy or radiotherapy before surgery. Tumor samples and the corresponding noncancerous mucosal tissue were collected from all patients immediately after resection and were frozen in liquid nitrogen. Samples were stored in the Center Laboratory of Second Affiliated Hospital of Fujian Medical University for studies.

### Evaluation of serum ADMA levels

The serum ADMA levels were measured using the enzyme linked immunosorbent assay (ELISA) method (Immunodiagnostik, Bensheim, Germany), following manufacturer’s instructions. In this study, we examined serum from 115 patients with Gastric adenocarcinoma patients and 110 noncancerous cases were used as controls. Differences of serum ADMA levels between cancer and noncancerous cases were tested with 2-tailed t-test. Relations between variables were investigated by Pearson’s correlation test. 2 × 10^8^ of six human GC cell lines were also measured.

### Cell proliferation assay

Cell proliferation was assessed using the Cell Counting Kit-8 (CCK-8; Dojindo, Kuma-moto, Japan). The GC cells were seeded in 96-well plates at a density of 1000 cells per well and incubated at 37 °C, 5% CO2 for 1, 2, 3, 4, 5 days. 10 ml of CCK-8 solution was added into each well and incubated at 37 °C for 1 h. The absorbance at 450 nm was measured using a microplate reader.

### Cell migration and invasion assay

For the migration assay, 6 × 10^4^ cells in serum free media were placed into the upper chamber of an insert (8-mm pore size; BD Bioscience). For the invasion assay, the transwell insert was coated with Matrigel (BD Bioscience) and 6 × 10^4^ cells were plated onto the top of the coated filters. The medium containing 10% fetal bovine serum was placed to the lower chamber. After 24 h of incubation, the cells that did not migrate or invade through the transwell insert were removed with cotton swabs and then the insert was stained with 0.1% crystal violet, imaged, and counted using an a Qimaging Micropublisher 5.0 RTV microscope camera (Olympus).

### Wound-healing assay

8 × 10^5^ of GC cells were plated into 6-well plates and reached to 100% confluence. Wounds were scratched onto the monolayer of cells with a 10ul pipette tip. Then the cells cultured at 37 °C in 5% CO2 and the images were captured at 0, 36 h.

### Western blot assay

Western blot was performed to analyze the expression of proteins. The following antibodies were used for this analysis, E-cadherin antibody, Vimentin antibody, β-catenin antibody, Snai antibody, Slug antibody, Twist antibody, (Cell Signaling Technology, Beverly, MA). Protein expression was quantifed by densitometric analysis, and the expression levels were normalization against that of β-actin (Sigma Aldrich).

### RNA extraction and real-time quantitative PCR

Total RNA were isolated from cell lines or frozen tissues with the Qiagen RNeasy kit as described by the manufacturer, and 1 mg RNA was reverse transcribed using miScript Reverse Transcription Kit (Qiagen, Hilden, German) for first strand complementary DNA synthesis. Quantitative PCR was performed using SYBR Premix EX Taq kit (Takara, Shiga, Japan). The specifc primers used were designed to detect the mRNA expression of E-cadherin, Vimentin, Twist, Slug and Snail. β-actin was including as an internal control. The comparative threshold cycle (Ct) method was used to determine the relative level of gene expression. Primers used for qRT-PCR analysis of EMT related markers and β-actin were listed in the following:

E-cadherin: 5′-GTCTGTCATGGAAGGTGCT-3′ (forward)

5′-TACGACGTTAGCCTCGTTC-3′(reverse)

Vimentin: 5′-CCACGAAGAGAAATCCAGG-3′ (forward)

5′-CAGAGAGGTCAGCAAACTTGG-3′(reverse)

Snail: 5′-CCTTCTCTAGGCCCTGGCT -3′ (forward).

5′-AGGTTGGAGCGGGTCAGC-3′ (reverse)

slug: 5′-ATCACAGTGACAGATGTCA-3′ (forward)

5′-AACGCAGTGTACAGAATCAG-3′ (reverse)

Twist: 5′-GGGCCGGAGACCTAGATG-3′ (forward)

5′-TTTCCAAGAAAATCTTTGGCATA-3′(reverse)

β-actin: 5′-CCTGGCACCCAGCACAAT-3′ (forward)

5′-GGGCCGGACTCGTCATACT -3′ (reverse)

### Immunohistochemistry and scoring methods

We constructed tissue microarrays, which include GC tissues and corresponding non-cancerous tissues extracted from 115 GC patients. Tissue microarray chip were included two cores of 1 mm diameter per sample. Immunohistochemical staining with β-catenin antibody (1:100, CST). Nuclear/cytoplasmic β-Catenin staining was considered positive if > 30% of cells showed yellow or brown staining.

### Dual-luciferase reporter assay

The TCF-responsive luciferase construction of Top-Flash and its mutant Fop-Flash (Addgene, USA) were used to study the β-catenin transcriptional activity. The GC cells were seeded into 24-well plates. After 12 h incubation, target cells were co-transfected with Top-Flash and PRL-TK report vector or Fop-Flash and PRL-TK report vector. The relative luciferase activity was determined using a dual-luciferase reporter assay kit (Promega, USA). The PRL-TK report vector was used as an internal control vector.

### Statistical analysis

Statistical analysis was examined using SPSS 22.0 for Windom. Student’s *t* test was used to analyze the results expressed as mean ± SD from three independent assays. The associations between the level of ADMA and the clinicopathological parameters of the GC patients were analyzed using the *χ*^2^ test or Fisher’s exact test. The survival curves were plotted using Kaplan–Meier analysis. Differences were considered significant when *P* < 0.05.

## Results

### The serum ADMA levels were higher in gastric carcinoma and inversely correlated with prognosis

**Fig. 1:**
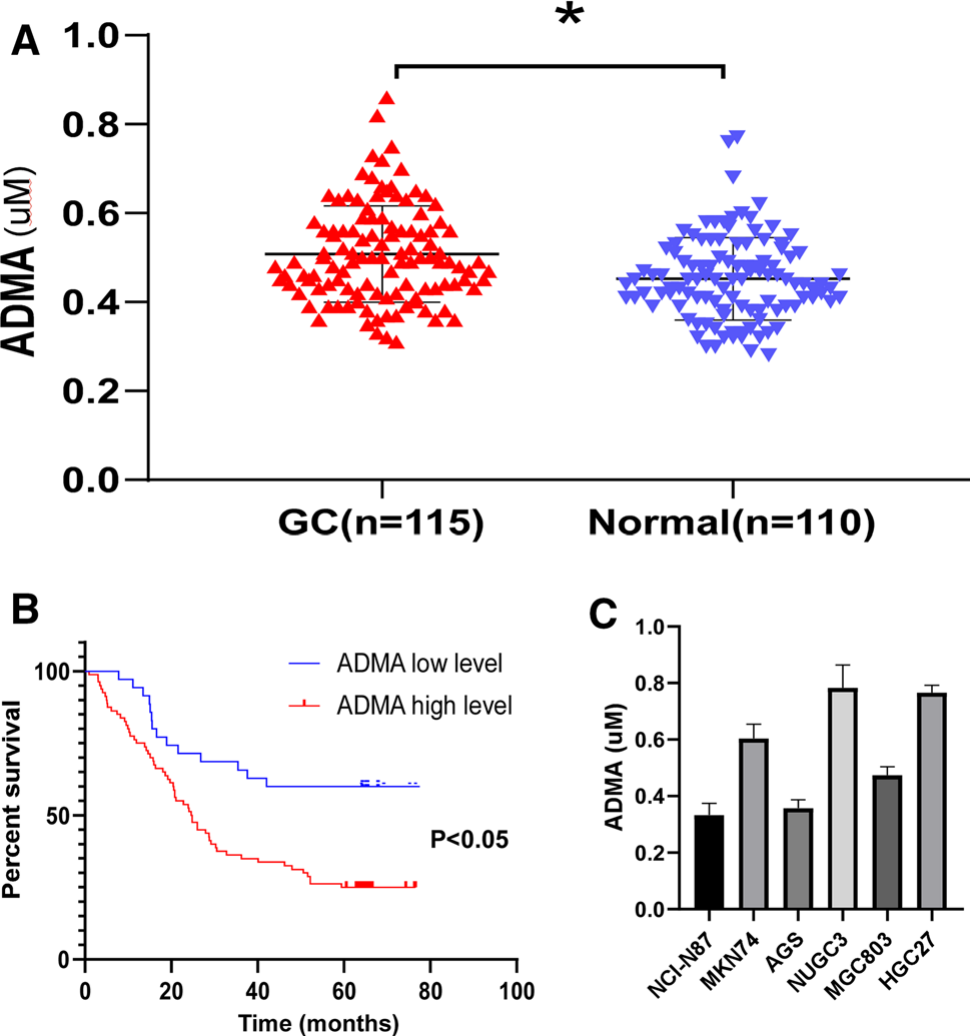
The serum ADMA levels were higher in gastric carcinoma and positively correlated with poor prognosis. **a** The serum ADMA levels in 115 GC patients before surgery and 110 normal subjects. Elisa test was carried out to determine the level of serum ADMA. The graph showed the concentrations of the serum ADMA levels in GC and normal subjects. Unpaired T test was used to compute P value (*P < 0.05). **b** Kaplan–Meier survival analysis of 115 GC patients with the high or low serum ADMA levels (*P *< 0.05, log-rank test). **c** the serum ADMA levels in six GC cell lines

We first measured serum ADMA level in 115 GC patients and 110 normal subjects using ELISA. As shown in Fig. 1a, the serum ADMA levels in patients with GC were significantly higher than normal subjects (*P *< 0.05). According to our result, the average of serum ADMA in the normal subjects was 0.447 μM (μM meaning μmol/L), so we defined GC patients with ADMA more than 0.447 μM as ADMA high level group, otherwise, as ADMA low level group. Then correlation analysis of serum ADMA levels with patients clinicopathological characteristics (Table 1) demonstrated that the ADMA level was positively associated with the depth of tumor invasion (*P* = 0.004) and clinical stage (*P* < 0.001), and negatively with differentiation status (*P* < 0.001). And serum ADMA level presented no significant association with gender, age, tumor size and lymph node metastasis. Kaplan–Meier survival analysis showed that GC patients with high level of ADMA had observably shorter survival than low level ADMA group (Fig. 1b). In summary, these results indicated that ADMA may function as a tumor activator and it may promote the development and progression of GC.

**Table 1:** Clinicopathologic characteristics of 115 GC patients according to the serum ADMA level

Variant	Total	ADMA level		*P* value
		Low	High	
Age (years)				0.685
< 60	72	16	56	
≥ 60	43	19	24	
Gender				0.226
Male	84	28	56	
Female	31	7	24	
Tumor size (cm)				0.125
< 5	70	25	45	
≥ 5	45	10	35	
Depth of tumor invasion				0.004*
T2	9	7	2	
T3	14	5	9	
T4	91	23	69	
Lymph node metastasis				0.080
N0	22	11	11	
N1	23	7	16	
N2	20	7	13	
N3	50	10	40	
Clinical stage				0.001*
I/II	28	16	12	
III/IV	87	19	68	
Differentiation				0.001*
High	8	7	1	
Middle	16	5	11	
Low/undifferentiation	91	23	68	

*Significant difference

### ADMA did not affect the proliferation, but promoted migration and invasion potential of GC cell

**Fig. 2:**
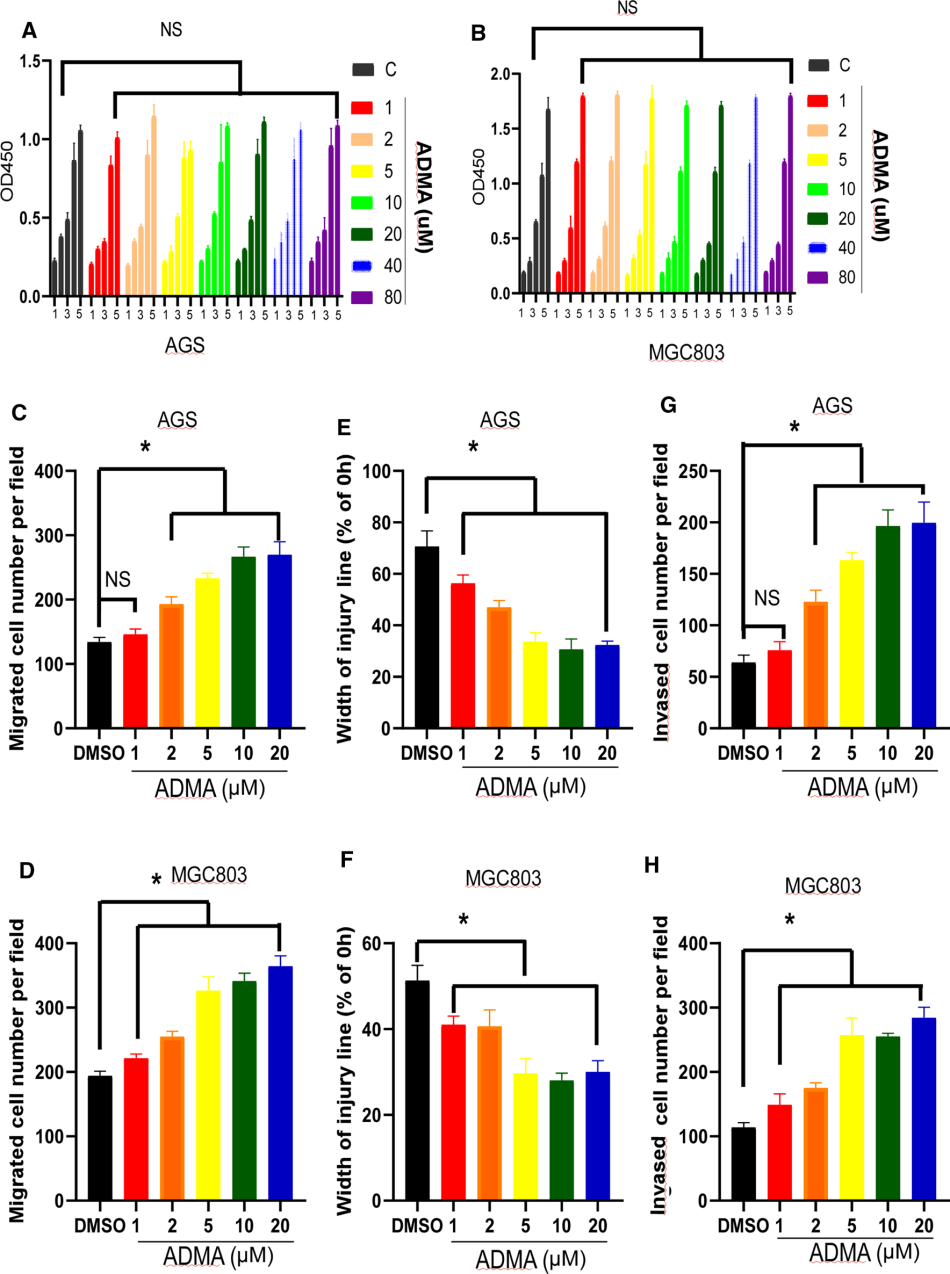
ADMA regulated GC cell migration and invasion but had no treatment on growth in vitro. **a**, **b** CCK8 assay demonstrating the influence of different concentrations of ADMA on the proliferation rate of AGS and MGC803 cells. Data were expressed as mean ± SD of three independent experiments (NS indicates no statistical significance). **c**, **d** Relative migration of the AGS and MGC803 through an uncoated filter toward serum-containing medium in a Boyden chamber assay. Data were expressed as mean ± SD of three independent experiments (*P < 0.05). **e**, **f** Relative motility as determined by the ability of AGS and MGC803 to close a wound made by creating a scratch through a lawn of confluent cells. Data were expressed as mean ± SD of three independent experiments (*P < 0.05). G and H, Relative invasion of the GC cells (AGS and MGC803) through a layer of Matrigel coated on the filter of a Boyden chamber. Data were expressed as mean ± SD of three independent experiments (*P < 0.05)

Based on the finding that ADMA level was associated with prognosis of GC, we determined the functional role of ADMA in GC malignant behaviors in vitro. First, we measured ADMA level in six cell lines (Fig. 1c). According to the level of ADMA, we chose AGS and MGC803 as objects of our following research. As showed in Fig. 2a and b, AGS and MGC803 were treated with different concentrations of ADMA(0, 1, 2, 5, 10, 20, 40, 80 μM), and we found ADMA did not affect the proliferation rate of AGS or MGC803 cells as evaluated by CCK-8 assay. As shown in Fig. 2c,2D and Supplementary Fig. 1A, migration assay showed that AGS and MGC803 were treated with different concentrations of ADMA (0, 1, 2, 5, 10, 20 μM), and ADMA enhanced the cell migratory ability. At the same time, a wound-healing assay was used to confirm the changes in cell migration. ADMA remarkably promoted the wound closure than the control cells (Fig. 2e, f and Supplementary Fig. 1B). And next, the invasive potential of the GC cells was assessed by a modified Boyden chamber invasion assay. Likewise, ADMA increased the number of cells that invaded through Matrigel compared with the control cells (Fig. 2g, h and Supplementary Fig. 1c). Taken together, these results clearly suggest that the important role of ADMA in GC metastasis. It is worth mentioning that ADMA(10μΜ) is optimum concentration. In the above mentioned experiments, as the concentration of ADMA increases from 0 to 10 μM, the experimental results become more obvious. While the ADMA concentration was higher than 10 μM, the experimental results were similar. Therefore, we chose ADMA (10 μM) as experimental group in the following assays.

### ADMA enhanced epithelial-mesenchymal transition (EMT)

**Fig. 3:**
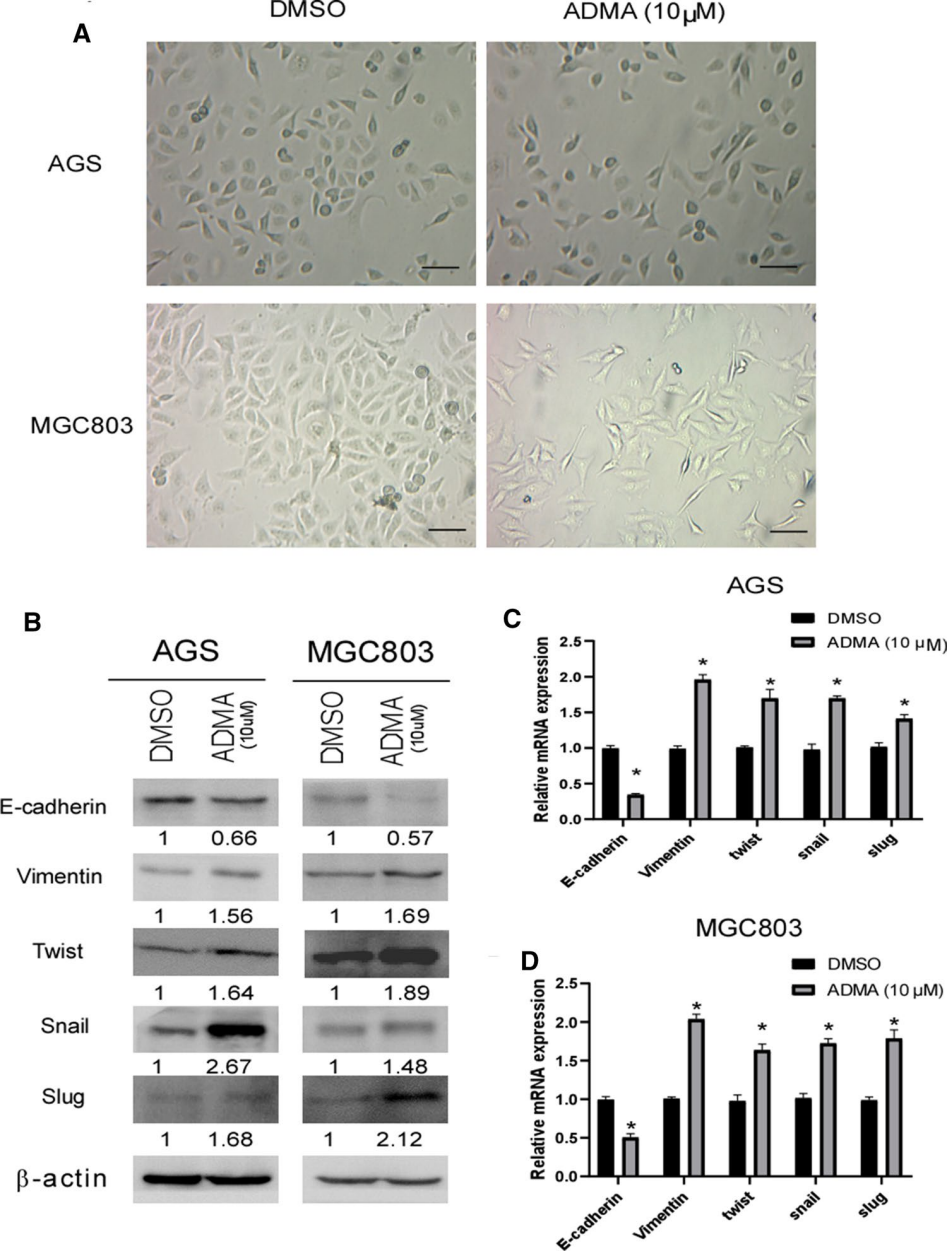
ADMA regulated the expression of EMT markers in the GC cells. **a** Bright field images of the morphology of AGS and MGC803 cells with the medium in DMSO and ADMA (10 μM) separately. Scale bar, 50 μm. **b** Western blot analysis illustrating the effect of ADMA(10 μM) on expression of EMT markers in AGS and MGC803 cells. **c**, **d** qRT-PCR analysis presenting the effect of ADMA (10 μM) on expression of EMT markers in AGS and MGC803 cells. The results were representative of three independent experiments (*P < 0.05)

Many studies have shown that EMT is considered a key event in the initial invasion step of cancer metastasis. which allows polarized epithelial cells to become mesenchymal cells. After a series of biochemical changes that induce a morphological transformation, epithelial cells reduced intercellular adhesion and enhanced migratory and invasive capabilities. As shown in Fig. 3a, bright field images of the morphology of GC demonstrated that ADMA (10 μM) endowed the GC cell with more fibroblast-like morphological features. Then Western blot analysis and qRT-PCR were used to quantify the effect of ADMA on protein and mRNA expression of EMT-related markers in GC cell lines. ADMA (10 μM) down-regulated the expression of epithelial markers (E-cadherin), while up-regulated the expression of mesenchymal markers (Vimentin) and EMT regulator (Snail, Slug and Twist) in protein and mRNA level (Fig. 3b–d).

### ADMA positively regulated β-catenin expression and promoted migration and invasion via Wnt/β-catenin pathway in GC

**Fig. 4:**
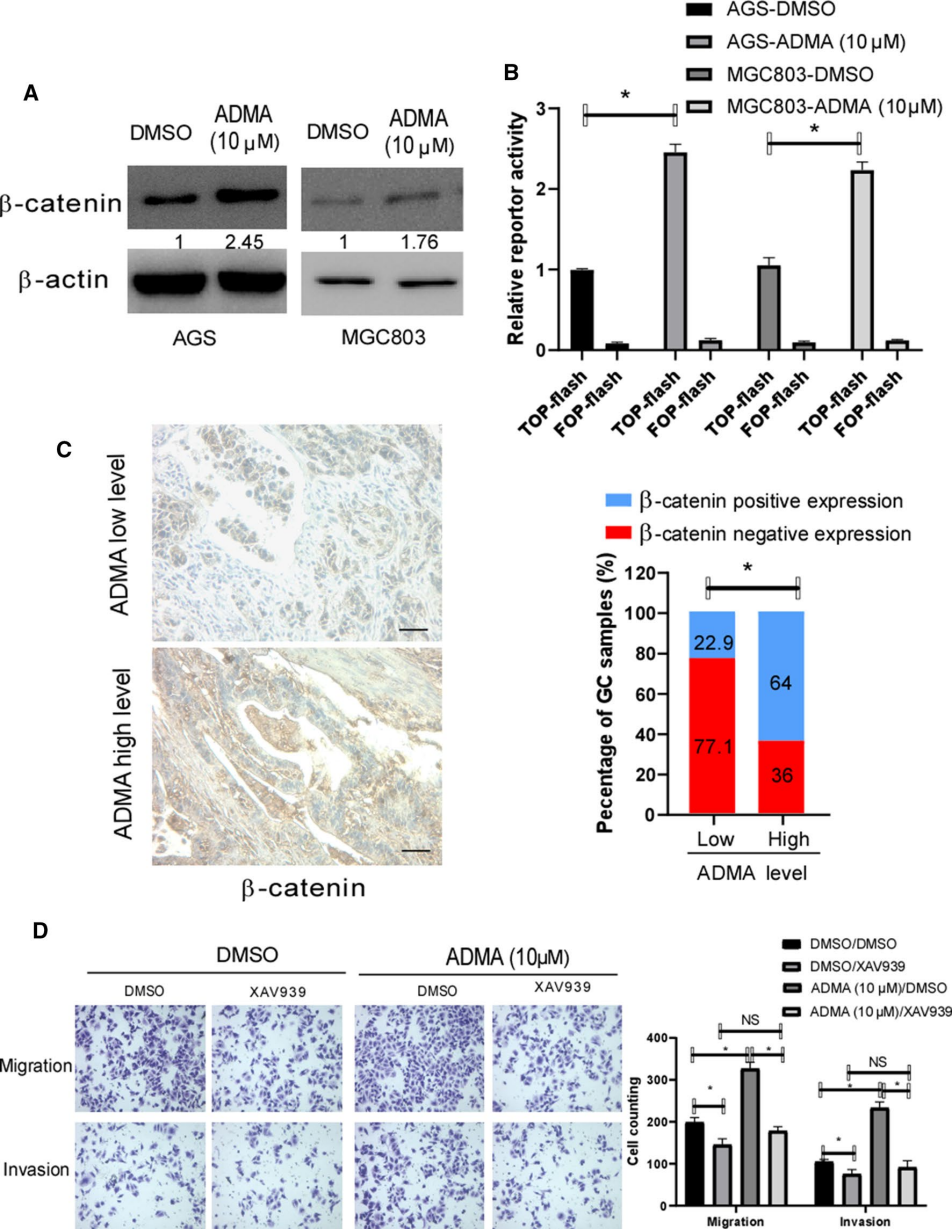
ADMA positively regulated β-catenin expression in GC and mediated through WNT signaling pathway. **a** Western blot demonstrated that ADMA (10 μM) enhanced the protein levels of β-catenin in AGS and MGC803. β-actin served as a loading control. **b** The ADMA(10 μM) in GC cell positively correlated with β-catenin activity in TOP-Flash reporter assay. Expression was normalized with Renilla luciferase activity. The experiments were performed three times independently(*P < 0.05). **c** IHC assay showing the relationship between serum ADMA level and β-catenin in the 115 GC samples. Scale bar, 50 μm. In the GC samples with high ADMA level, the percentage of β-catenin positive expression was 64%, significantly higher than those with low ADMA level (22.9%) (*P < 0.05). **d** The stimulatory effect of ADMA on the GC cell migration and invasion blocked by WTN inhibitor XAV939, as the representative images were presented (*P < 0.05; NS, no statistical significance)

Wnt/β-catenin pathway is well known to play an important role in inducing EMT and promoting migration and invasion of GC. To investigate whether ADMA affected β-catenin expression in GC, As shown in Fig. 4a, ADMA level was positively correlated with the expression of β-catenin in AGS and MGC803. Then, to investigate whether ADMA affected β-catenin transcriptional activity in GC, Top-Flash reporter assays were used to assess the effect of ADMA on β-catenin activity. As showed in Fig. 4b, ADMA significantly increased the transcriptional activity of β-catenin in AGS and MGC803. To further confirm the positive correlation of ADMA and β-catenin expression. As shown in Fig. 4c, IHC assay showed ADMA level was closely correlated with the expression of β-catenin. Given the full evidence that ADMA positively regulated β-catenin expression in AGS and MGC803, we speculated that one route by which high level ADMA promotes GC migration and invasion may be through activation of β-catenin. To explore this, we transiently used WNT inhibitor to treat the GC cells, then measured the effect of pharmacological inhibition on the AGS migration and invasion. As showed in Fig. 4d, treatment of GC cells with WNT inhibitor XAV939 abrogated the effect of ADMA enhanced cell migration and invasion. Given to the data, we can make the conclusion that the effect of ADMA on GC migration and invasion is mediated primarily by the enhanced β-catenin.

## Discussion

The central observation of this report is that the level of serum ADMA is higher in GC samples and its high level is closely related to depth of tumor invasion, clinical stage, poor differentiation and the poor clinical outcome. In our study, serum ADMA level was elevated in patients with GC and high concentration of AMDA in GC cells enhanced cell migration and invasion in vitro via Wnt/β-catenin pathway. Given to these evidences, we speculated that ADMA functions as a tumor activator in GC and may benefit clinical practice.

We found that ADMA did not make any effects to the rate of proliferation in vitro. Li et al. also showed that ADMA treatment did not impact the proliferation rate of LoVo cells, which was similar to our findings [15]. According to the clinical data, we found serum ADMA level is positively correlated with depth of tumor invasion and clinical stage. Given to these evidences aforementioned, we speculated that ADMA may be associated with the migration and invasion of GC cell. Then, we performed an analysis of cells migration and invasion using Boyden two chamber assay and wound-healing/scratch assay. In our study, we found ADMA increased the motility and invasion of GC cell. There is no doubt that it is the first time to report that ADMA increased GC cell motility and invasion.

In our present study, we also found ADMA induced morphological changes of GC. ADMA treatment caused that GC lost their epithelial cobblestone-like morphology to acquire a more elongated fibroblast-like shape. The result appeared to the theory of epithelial-mesenchymal transition (EMT). The EMT of GC, a process by which epithelial cells lose their orientation and cell–cell basement membrane contacts and then acquire mecenchyme features, contributes to invasion and cancer progression of cancer[21, 27, 28]. Therefore, it is tempting to speculate that ADMA enhance the migration and invasion in GC media EMT. Furthermore, a series of experiments were performed. We discovered that the expression of E-cadherin was significantly reduced in GC cell with ADMA. Inversely, the expression of mesenchymal markers (Vimentin) and transcription factors (snail, slug and twist) were up-regulated. According to some studies, EMT is activated by a number of transcription factors, including Snail, Slug, and Twist, and also by the repression of E-cadherin expression [29, 30]. One of the most common features of EMT is the loss of E-cadherin expression. Snail and Slug have been reported to be associated with tumor cell migration and invasion. As a key regulator of EMT, Snail was first discovered in Drosophila as a zinc-finger transcription factor and repressed E-cadherin transcription by binding to the E-box site in the promoter of E-cadherin [31, 32]. Slug belongs to the Slug family of zing-finger transcription factors and plays a major role in EMT during embryonic development and metastasis of various cancers by inhibiting E-cadherin [33]. Thus, our findings indicated the possible role of Snail/Slug associated EMT in the pathogenesis and development of GC, which was associated with ADMA. Twist was a basic helix– loop–helix domain-containing transcription factor and a highly conserved protein which can suppress apoptosis, whose functions include inducing EMT and enhancing migration and invasion of tumor cells, inhibiting cell apoptosis, promoting tumor angiopoiesis, as well as causing chromosome instability. Some studies have reported the mechanism underlying gene transcriptional activation by Twist [34–36]. Likewise, the results of Cao’s study suggested that Snail and Twist work synergistically to induce EMT [34]. In conclusion, ADMA induces GC cell migration and invasion in vitro likely by means of activation of an EMT process.

We next aimed to identify the signaling mechanism by which ADMA mediates migration and invasion in GC cells. Additionally, it had reported that loss of DDAH1 in GC promoted the EMT progression as well as the migration and invasion via Wnt signaling pathway. Similarly, DDAH1 inhibitor (PD404182) came to the same result [19]. ADMA are mainly hydrolyzed by DDAH1 [37]. DDAH1 inhibitor (PD404182) treatment resulted in the accumulation of ADMA in vitro. So we speculated whether ADMA promoted migration and invasion of GC cell via Wnt signaling way as well. Therefore, a series of assays were performed to support our guess. In our studies, western blot analysis and dual-luciferase reporter assay manifested that ADMA significantly increased the level and the transcriptional activity of β-catenin in GC cell. To further confirm the positive regulation of ADMA on β-catenin expression, immunohistochemical staining was performed on the GC tissues. The result is indicative of the closely relationship between ADMA and the expression of β-catenin. Given the full evidence that ADMA positively regulated β-catenin expression in GC. We speculated that one route by which ADMA promotes migration and invasion of GC may be through activation of β-catenin. Consequently, we found that treatment with a specific Wnt inhibitor (XAV939) abrogated the stimulatory effect of ADMA on migration and invasion in the GC cells. Together, these present results support a concept that ADMA mediates migration and invasion of GC cells via Wnt/β-catenin signaling pathway.

In summary, this is the unprecedented study exploring the effect of ADMA in GC and demonstrate for the first time that ADMA is likely to act as a tumor activator in GC. GC patients with high serum ADMA level is strongly correlated with tumor progression and clinical prognosis. With the development of medical technology, we are able to apply it to clinical practice as a diagnostic and prognostic biomarker. In addition, it is difficult to regulate the level of ADMA in tumor until now, but we offer insights into the therapy of GC.

## Electronic supplementary material

Below is the link to the electronic supplementary material.

Supplementary file1 Figure supplementary 1: A, Relative migration of the AGS and MGC803 through an uncoated filter toward serum-containing medium in a Boyden chamber assay. B. Relative motility as determined by the ability of AGS and MGC803 to close a wound made by creating a scratch through a lawn of confluent cells. C. Relative invasion of the AGS and MGC803 through a layer of Matrigel coated on the filter of a Boyden chamber. (JPG 3114 kb)
