# Supplementary figures and images for: ADMA mediates gastric cancer cell migration and invasion via Wnt/β-catenin signaling pathway
Source: Clin Transl Oncol. 2020 Jun 30;23(2):325–34. doi: 10.1007/s12094-020-02422-7 (PMC7854427; doi:10.1007/s12094-020-02422-7)

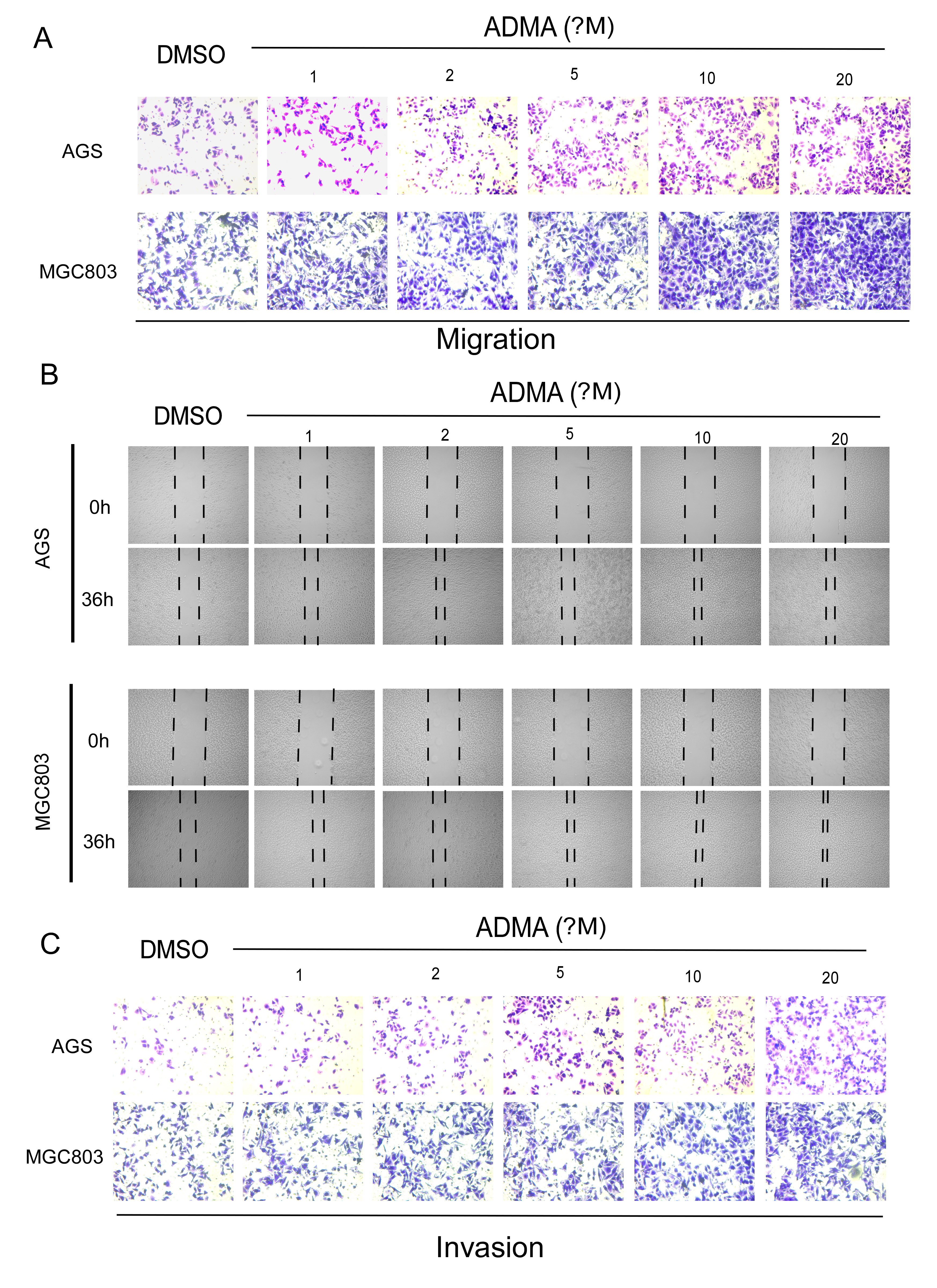

Supplement: Supplementary file 1 — Supplementary file1 Figure supplementary 1: A, Relative migration of the AGS and MGC803 through an uncoated filter toward serum-containing medium in a Boyden chamber assay. B. Relative motility as determined by the ability of AGS and MGC803 to close a wound made by creating a scratch through a lawn of confluent cells. C. Relative invasion of the AGS and MGC803 through a layer of Matrigel coated on the filter of a Boyden chamber. (JPG 3114 kb) [file 12094_2020_2422_MOESM1_ESM.jpg]
